# Polygenic Score Identifies Athletes at Increased Risk for Slower Recovery After Sport-Related Concussion: A Concussion Assessment, Research, and Education (CARE) Consortium Study

**DOI:** 10.1007/s40279-026-02477-6

**Published:** 2026-06-30

**Authors:** Zhiqi Zhang, Jill L. Reiter, Kelly N. H. Nudelman, Dongbing Lai, Thomas W. McAllister, Michael A. McCrea, Steven P. Broglio, Paul F. Pasquina, Jie Ren, Yunlong Liu, CARE Consortium Investigators, Louise A. Kelly, Justus Ortega, Nicholas Port, Margot Putukian, Jane McDevitt, Kevin Michael Bernstein, Darren Campbell, John DiFiori, Christopher D’Lauro, Kenneth L. Cameron, Adam Susmarski, Chris Giza, Joshua T. Goldman, Holly J. Benjamin, Thomas Buckley, Thomas Kaminski, James R. Clugston, Joseph B. Hazzard, Patrick G. O’Donnell, Luis A. Feigenbaum, James T. Eckner, Jason P. Mihalik, Christina L. Master, Micky Collins, Anthony P. Kontos, Alison Brooks, Stefan M. Duma, Steve Rowson, Laura J. Lintner, Christopher M. Miles

**Affiliations:** 1 Center for Computational Biology and Bioinformatics, Indiana University School of Medicine, Indianapolis, IN USA; 2 Department of Biostatistics and Health Data Science, Indiana University School of Medicine, Indianapolis, IN USA; 3 Richard M. Fairbanks School of Public Health, Indianapolis, IN USA; 4 Department of Medical and Molecular Genetics, Indiana University School of Medicine, Indianapolis, IN USA; 5 Department of Psychiatry, Indiana University School of Medicine, Indianapolis, IN USA; 6 Department of Neurosurgery, Medical College of Wisconsin, Milwaukee, WI USA; 7 Michigan Concussion Center, University of Michigan, Ann Arbor, MI USA; 8 Department of Physical Medicine and Rehabilitation, Uniformed Services University of the Health Sciences, Bethesda, MD USA

## Abstract

**Background:**

Emerging evidence suggests that genetic variations contribute to individual differences in concussion recovery. A polygenic score (PGS) can capture the cumulative effect of multiple genetic variants, providing a partial explanation for individual differences in recovery times.

**Objectives:**

In this study, our primary objective was to investigate the association between a PGS and recovery time following sport- or military activity-related concussion to better understand the genetic influences on recovery and inform personalized treatment strategies.

**Methods:**

Genome-wide genotyping data from collegiate athletes and military cadets with confirmed sport- or military activity-related concussions were obtained from the CARE Consortium study (*N* = 4659). A genome-wide association study (GWAS) was conducted on the discovery cohort (*n* = 768 samples) to identify single-nucleotide polymorphisms (SNPs) associated with time to return-to-play initiation (RTPi) after concussion using log-logistic accelerated failure time (AFT) models, adjusted for demographic and injury-related factors, and the first five principal components of genetic ancestries. Linkage disequilibrium-clumped SNPs with the lowest *p* values were selected and further refined using a robust variable selection method under a multivariable AFT model, resulting in the final set of SNPs. The PGS was constructed from this final set of SNPs and validated in an independent validation cohort (*n* = 262 samples) for time to RTPi. The validation was extended to the biologically relevant slow RTPi outcome (≥ 14 days) using a logistic regression model. We also tested the PGS for association with time to return-to-play (RTP) and slow RTP (≥ 28 days) as secondary outcomes. A Gene Ontology (GO) enrichment analysis was performed on the selected SNPs to identify relevant biological processes.

**Results:**

The PGS was constructed using 49 SNPs. We found that higher PGS was significantly associated with a longer time to RTPi after concussion (*p* = 0.001), where each standard deviation increase in the PGS was associated with a 12% longer time to RTPi. The PGS was significantly associated with a slow RTPi recovery (≥ 14 days), with each standard deviation increase corresponding to an 82% higher odds of delayed recovery (*p* = 0.002). No significant associations were observed between PGS and RTP outcomes. GO enrichment analysis highlighted synaptic mechanisms related to organization, signaling, and neuronal repair, which are critical for restoring neural communication during the healing process.

**Conclusions:**

Higher PGS is significantly associated with longer recovery time after concussion. This finding provides novel genetic insights for developing personalized treatment strategies to reduce the burden of prolonged recovery from concussion injury.

**Supplementary Information:**

The online version contains supplementary material available at https://doi.org/10.1007/s40279-026-02477-6.

## Key Points

**Table:** 

A person’s genetics can influence how long it takes them to recover from a concussion.
This study identified genetic pathways related to brain cell communication and repair that may play a role in concussion recovery.
These insights may help guide future efforts to create more personalized concussion care and support for athletes.

## Introduction

Concussion is a common form of mild traumatic brain injury (mTBI), particularly prevalent among athletes and military personnel, with recovery trajectories varying widely between individuals [1, 2]. Clinically, mTBI is generally defined by a Glasgow Coma Scale (GCS) score of 13–15 [3, 4]. While most individuals achieve clinical recovery within days to weeks, a subset experiences prolonged symptoms, leading to disruptions in physical, cognitive, and emotional functioning [5–7]. Identifying predictors of recovery is crucial for guiding clinical care and tailoring interventions to improve outcomes [8]. Multiple factors influence recovery from concussion, including biological sex, injury severity, concussion history, post-injury symptom burden, and immune-inflammatory responses [5, 6, 9, 10]. However, despite advances in understanding these influences, the contribution of genetic factors remains largely unexplored.

Genome-wide association studies (GWAS) are valuable tools for investigating genetic variants in mTBI research, and prior GWAS have identified variants associated with TBI/concussion susceptibility [11]. However, evidence for the genetics of recovery after concussion remains limited, representing an important gap that warrants further investigation. Emerging evidence suggests that genetic variations contribute to individual differences in recovery outcomes of mTBI [12, 13]. For example, the catechol-*O*-methyltransferase *(COMT)* Val158Met polymorphism has been associated with better preservation of nonverbal processing speed at 6 months following mTBI [12]. Similarly, the brain-derived neurotrophic factor *(BDNF)* Val66Met polymorphism has been linked to reduced cognitive processing speed following mild-to-moderate TBI [13]. These findings suggest genetic predispositions may influence recovery trajectories. However, prior studies were primarily focused on single-gene or single-nucleotide polymorphism (SNP) association, which failed to fully capture the cumulative effects of genetic risks.

A polygenic score (PGS), which aggregates the effects of multiple SNPs into a single genetic risk measure, offers a promising approach to evaluate the overall genetic liability of a condition and has shown utility in predicting outcomes across various conditions [14–17]. However, research on PGS for concussion-related outcomes remains limited. Antrobus et al. constructed a total genotype score from eight candidate polymorphisms to distinguish elite rugby players from nonathletes [18]. More recently, Dybing et al. applied an Alzheimer’s disease PGS to Concussion Assessment, Research, and Education (CARE) Consortium athletes and observed a nominal association with return to play (RTP) time in the < 24-days subgroup, which did not persist after covariate adjustment [19]. To the best of our knowledge, no study has derived and validated a PGS specifically for concussion recovery outcomes.

To fill this gap, we developed and tested a concussion-related PGS by utilizing data from the CARE Consortium [20]. We focused on the first recovery metrics collected in the CARE study, specifically time to return-to-play initiation (RTPi) and slow RTPi. RTPi marks the point at which a concussed individual is medically cleared to begin the graduated return-to-play (RTP) protocol [5]. Prolonged RTPi is clinically meaningful since affected individuals may face extended functional impairment, delayed return to activities, and increased risk of persistent symptoms. The CARE Consortium dataset provides a unique opportunity to evaluate genetic contributions to recovery trajectories while accounting for potential confounding factors, such as injury setting, concussion history, and symptom severity. We first constructed a PGS in a discovery cohort, then validated it in a separate, independent cohort. Both cohorts were analyzed for time-to-RTPi outcomes, with validation extended to the biologically relevant slow RTPi outcome. In addition, unrestricted RTP signifies when individuals resume full activity following a concussion. Therefore, RTP outcomes were assessed in a secondary analysis. Our study aimed to advance the understanding of genetic influences on concussion recovery while highlighting the potential of PGS as a predictive tool for personalized concussion management.

## Methods

### CARE Consortium and Study Participants

The CARE Consortium study is a 30-site, large-scale prospective cohort study supported by the National Collegiate Athletic Association (NCAA) and the U.S. Department of Defense, designed to examine the natural history of concussion in collegiate athletes and military cadets. Detailed descriptions of participant recruitment, concussion assessments, and recovery protocols of the CARE study have been reported previously [20]. In brief, participants underwent preseason baseline assessments before their sport season or academic year, during which demographics, medical history, and health measures were collected. Concussion cases were identified prospectively by team medical staff, and each diagnosis was confirmed by physicians using a common concussion definition [21] across all CARE sites. For concussed participants, post-injury follow-up assessments were conducted at five key time points, including immediately after injury (within approximately 6 h), 24–48 h post-injury, at RTPi, at unrestricted RTP, and at 6 months post-injury. In this study, slow RTPi was defined as taking 14 days or longer (≥ 14 days), while typical RTPi was defined as less than 14 days (< 14 days). This definition was established based on previous CARE studies, in which approximately 80% of participants achieved RTPi by 14 days [5, 6]. Slow RTP was defined as 28 days or more (≥ 28 days), whereas typical RTP occurred in under 28 days (< 28 days) [6]. Concussion symptoms were assessed using the Sport Concussion Assessment Tool (SCAT), which includes a 22-item symptom inventory, with severity of each symptom rated on a 0–6 scale [5, 20]. The SCAT symptom severity score was determined at the preseason baseline and 24–48 h post-injury to account for individual differences in symptom burden. Injury setting includes three categories: practice/training, competition, and outside sport. The ‘outside sport’ category includes military activity–related injuries.

**Fig. 1:**
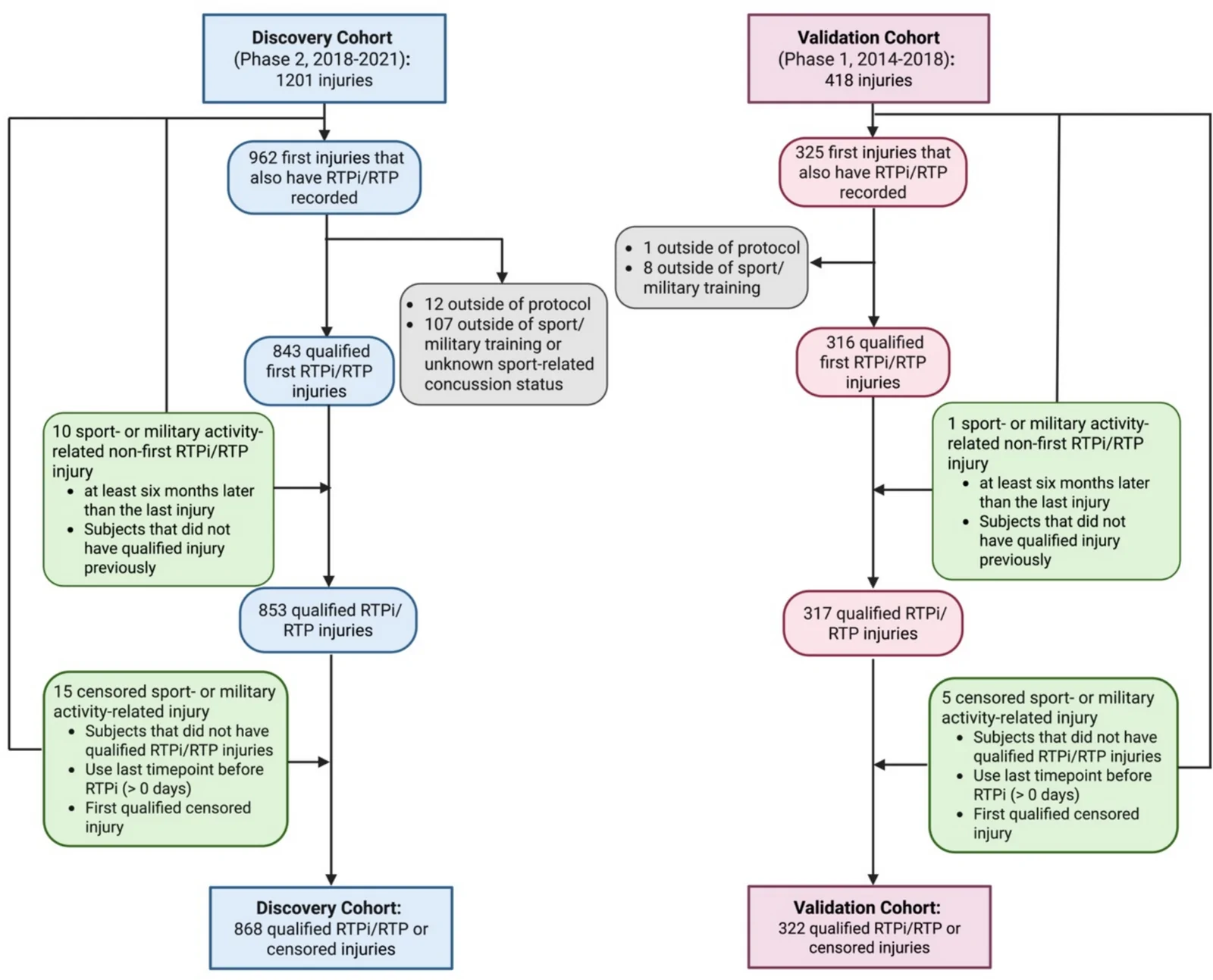
CONSORT (Consolidated Standards of Reporting Trials) diagram of participants in the discovery and validation cohorts. Created in BioRender. Zhang, Z. (2026) https://BioRender.com/q59p456. *RTPi* return to play initiation, *RTP* return to play

A CONSORT (Consolidated Standards of Reporting Trials) diagram of the study participants is provided in Fig. 1. The discovery and validation cohorts were defined based on separate genotyping batches within the CARE Consortium. The validation cohort consisted of participants enrolled in the CARE Consortium phase 1 (2014–2018), and the discovery cohort consisted of participants enrolled in the CARE Consortium phase 2 (2018–2021) [22]. The earliest concussion event recorded for participants during the study period was referred to as the first injury. For participants with multiple concussions, if the first injury was not eligible for inclusion, a later qualifying injury was included, provided it occurred at least 6 months after the previous concussion. Concussions not related to sport or military activities were excluded from the study. For concussion cases missing RTPi but with available RTP, the time to RTPi was imputed by subtracting the median RTP protocol duration (6.0 days) from the recorded time to RTP. The median RTP protocol duration represents the median time required to progress through the RTP protocol following RTPi [5]. For participants missing both RTPi and RTP, the time of the 24–48 h post-injury assessment was used as the right-censoring time in the accelerated failure time (AFT) model, as it represents the last known follow-up without an observed recovery. A total of 868 and 322 qualified sport- or military activity-related concussions were included in the discovery cohort and validation cohort, respectively. After excluding injuries with missing data on covariates (injury setting, preseason baseline SCAT symptom severity score, and post-injury 24–48-h SCAT symptom severity score) or a time to RTPi < 48 h, the overall missing rate for all covariates and RTPi < 48 h was 11.52% in the discovery cohort and 18.63% in the validation cohort. The final analytical samples included 768 participants in the discovery cohort and 262 participants in the validation cohort.

### Sample Collection Procedures

Six CARE sites participated in biospecimen collection for the validation study as part of the Advanced Research Core. Participants at these sites underwent venipuncture at preseason baseline and at all subsequent post-injury visits. Whole blood was collected in 10-mL tubes and centrifuged within 30 min of collection for 15 min at 1500 relative centrifugal force at 4° C to separate plasma and buffy coats. Samples were aliquoted into cryovials and stored at − 80° C, then shipped to the CARE Consortium biorepository at Indiana University School of Medicine for processing and storage. In the discovery cohort, 520 participants had saliva collected in Oragene saliva collection tubes (DNA Genotek, Inc., Kanata, Canada). Saliva samples were stored at room temperature, then shipped at ambient temperature to the CARE Consortium biorepository for processing and storage. DNA was extracted from buffy coats and saliva using standard operating procedures and sent to the Children’s Hospital of Philadelphia Center for Applied Genomics (CAG) for GWAS array data generation.

### Genotype Data Processing and Imputation

Genotype data were generated for 4659 participants, regardless of injury status, in two batches: CARE phase 1 (*N* = 551) and CARE phase 2 (*N* = 4108). Both batches were run on the Illumina Global Screening Array v2.0 BeadChip (Illumina, Inc., San Diego, CA, USA) at the Children’s Hospital of Philadelphia CAG following standard operating procedures (https://www.research.chop.edu/cag-genotyping-core) and the manufacturer-provided kit and instructions. The Illumina Global Screening Array v2.0 is a high-density genotyping microarray including over 700,000 SNPs genome-wide.

Data were aligned to genome build GRCh37/UCSC Human Genome 19. However, data were received and processed at different times, as described below. Principal components (PCs) of genetic ancestries were calculated using the R package *SNPRelate* [23], based on genotyped SNPs. Imputation was performed using the Michigan Imputation Server [24]. Pre-imputation quality control (QC) steps for both batches included strand alignment checks, removal of duplicate variants, and harmonization of variant identifiers. Since there was a time lapse between data processing of the first and second batches, different reference panels and updated workflows were used, which are described below.

#### Discovery Cohort

Using PLINK v1.9 [25], samples were removed for call rates < 98%, and variants were removed for call rates < 95%. Monomorphic variants and variants with Hardy–Weinberg equilibrium *p* values < 1 × 10^−6^ were excluded. Imputation was performed using the Michigan Imputation Server with the 1000 Genomes Phase 3 (v5) as the reference panel [26]. After imputation, PLINK v2.0 [27] was used with the default settings to convert the variant call format (VCF) files to plink format, with dosage = DS to hard-code dosage based on probabilities, and a similar QC process was followed. A second round of QC was then performed to exclude variants with imputation quality scores (*R*^2^) < 0.5, genotyping call rates below 95%, Hardy–Weinberg equilibrium *p* values < 1 × 10^−6^, and monomorphic variants. Genotyped individuals who were non-concussed participants were not relevant to this study and were not included in further analysis. The final set of genotyped samples in the discovery cohort consisted of 768 independent individuals. These samples were extracted, and variants with a minor allele frequency (MAF) < 5% were excluded.

#### Validation Cohort

Variants were filtered based on the following QC criteria: Hardy–Weinberg equilibrium *p* value < 1 × 10^−6^, MAF < 1%, and genotyping call rate < 95%. All samples with a genotype rate > 95% were retained. Imputation was performed via the Michigan Imputation Server using the Haplotype Reference Consortium (HRC) reference panel [28]. After imputation, variants with *R*^2^ < 0.5, genotyping call rates < 95%, Hardy–Weinberg equilibrium *p* values < 1 × 10^−6^, and monomorphic variants were excluded. Genotyped individuals who were non-concussed were excluded from further analysis. The final set of genotyped samples in the validation cohort consisted of 262 independent individuals; these samples were extracted, and variants with MAF < 5% were excluded. A total of 1,681,939 SNPs overlapping between the discovery and validation cohorts were included in the subsequent GWAS.

### PGS Construction

A GWAS was performed in the discovery cohort to identify SNPs associated with time to RTPi following concussion. The analysis utilized log-logistic AFT models because the proportional hazard assumption was severely violated in both the discovery and validation cohorts, as assessed by the Grambsch–Therneau test. AFT directly models survival time by assuming a parametric distribution for time-to-event data and estimating how predictors accelerate or decelerate the timing of the event [29]. We selected the log-logistic distribution over log-normal and Weibull distributions because it provided a better fit for these data. The models were adjusted for age at injury, sex, injury setting (competition, practice/training, or outside sport), diagnosed concussion history prior to the qualified CARE injury (≥ 2 vs ≤ 1 previous concussions), SCAT scores at baseline and 24–48 h post-injury, as well as the first five principal components (PCs) of genetic ancestries. Linkage disequilibrium (LD) clumping was performed with an *R*^2^ threshold of < 0.1 and a window size of 200 kb, retaining the SNP with the lowest *p* value within each window. The top 300 SNPs with the lowest *p* values (largest *p* value among the selected SNPs = 5.728 × 10^–4^) from the initial GWAS-based marginal screening were then jointly analyzed using a robust variable selection method under the AFT model [30], using the regnet function in the R package *regnet* [31]. The weighted least absolute deviation (LAD) estimator adopted in this method provided robustness to skewness in the survival data and mitigated the influence of outliers. By incorporating the Minimax Concave Penalty (MCP) with the LAD loss function, this method efficiently identified important SNPs associated with the survival outcome in high-dimensional genetic data. This process resulted in a final set of 49 SNPs that were used to construct the PGS. We refitted these 49 SNPs in a log-logistic AFT model with the same covariates and used the resulting coefficients as the weights to construct the PGS.

### PGS Validation

#### Time-to-Event Outcomes

The effects of the constructed PGS on time to RTPi and time to RTP were tested in the validation cohort using log-logistic AFT models adjusted for the same covariates used in the construction step. For ease of interpretation, we standardized the raw PGS prior to analysis by centering it around zero and dividing by the standard deviation. A higher (standardized) PGS corresponds to a longer time to reach RTPi and unrestricted RTP, reflecting slower recovery. Predictive performance was evaluated using the concordance index (C-index), which quantifies the model’s ability to correctly rank individuals by time-to-event outcomes.

#### Binary Outcomes

We further evaluated the effect of the PGS on slow recovery outcomes, defined as binary indicators of slow RTPi and slow RTP derived from their respective time-to-event measures. Slow RTPi was coded as 1 if RTPi occurred ≥ 14 days post-injury (0 otherwise), and slow RTP was coded as 1 if unrestricted RTP occurred ≥ 28 days post-injury (0 otherwise). Slow recovery outcomes were analyzed in the validation cohort using logistic regression models adjusted for the same covariates. Injuries missing both RTPi and RTP data were excluded from this analysis. The PGS was standardized prior to analysis for easier interpretation. Predictive performance was assessed using the area under the receiver operating characteristic curve (ROC-AUC), which is analogous to the *C*-index. The DeLong method was used to estimate 95% confidence intervals for AUCs and to perform pairwise comparisons of AUCs from correlated ROC curves across models in the validation cohort.

### Gene Ontology Enrichment Analysis

Gene Ontology (GO) enrichment analysis identifies biological terms significantly associated with a gene set by comparing observed annotations to a random background. The SNPs used for PGS construction were annotated using ANNOVAR [32] with the NCBI RefSeq GRCh37 reference genome, with intergenic SNPs assigned to their nearest gene. GO enrichment analysis was performed using the R package *clusterProfiler* [33], applying the Benjamini-Hochberg (BH) method to adjust for multiple testing, with a false discovery rate (FDR) < 0.05 considered significant. The analysis included all three GO categories: biological process, molecular function, and cellular component.

## Results

The descriptive statistics of study participants in the discovery (*N* = 768) and validation (*N* = 262) cohorts are summarized in Table 1. Sex by racial subgroup composition within the discovery and validation cohorts is further detailed in Supplementary Table 1 in the electronic supplementary material (ESM). Group comparisons were conducted using an ANOVA test for continuous variables and Fisher's exact test for categorical variables. The mean ages at injury (± SD) in the discovery and validation cohorts were 20.08 ± 1.28 and 20.02 ± 1.34, respectively. In both cohorts, the majority of participants were male, European American, had sustained injuries during practice or training settings, and had fewer than two previous concussions. The discovery cohort included more females than the validation cohort and had a higher proportion of participants who were injured during practice or training. Both cohorts exhibited similar preseason baseline SCAT symptom severity scores. However, the post-injury 24–48-h SCAT symptom severity score was higher in the discovery cohort compared with the validation cohort. Potential confounding effects of age, sex, injury setting, previous concussion counts, preseason baseline SCAT symptom severity scores, and post-injury 24–48-h SCAT symptom severity scores were controlled during PGS construction and validation. The proportion of participants classified as having slow RTPi (≥ 14 days) was similar between the discovery (24.14%) and validation (24.51%) cohorts. The proportion of slow RTP cases (≥ 28 days) was not statistically significantly different between the discovery (17.51%) and validation (13.62%) cohorts. The raw, calculated PGS before standardization (mean ± SD) in the discovery and validation cohorts was − 0.73 ± 0.48 and − 0.75 ± 0.43, respectively, with a nonsignificant *p* value of 0.45.

**Table 1:** Demographic characteristics of the discovery and validation CARE cohorts

	Discovery cohort (*N* = 768)	Validation cohort (*N* = 262)	*p* Value
Age at injury			0.502
Mean (SD)	20.08 (1.28)	20.02 (1.34)	
Sex			< 0.001
Male	452 (58.85%)	194 (74.05%)	
Female	316 (41.15%)	68 (25.95%)	
Race			0.074
European American	546 (71.09%)	167 (63.74%)	
African American	125 (16.28%)	56 (21.37%)	
Other	97 (12.63%)	39 (14.89%)	
Injury setting			< 0.001
Practice/training	497 (64.71%)	151 (57.63%)	
Competition	245 (31.90%)	82 (31.30%)	
Outside sport	26 (3.39%)	29 (11.07%)	
Previous concussion			0.276
< 2	706 (91.93%)	247 (94.27%)	
≥ 2	62 (8.07%)	15 (5.73%)	
SCAT at preseason baseline			0.977
Mean (SD)	6.65 (11.44)	6.68 (10.99)	
SCAT 24–48 h after injury			< 0.001
Mean (SD)	29.98 (20.85)	23.15 (18.99)	
Slow RTPi (≥ 14 days)			0.933
No	572 (75.86%)	194 (75.49%)	
Yes	182 (24.14%)	63 (24.51%)	
N/A	14	5	
Slow RTP (≥ 28 days)			0.173
No	622 (82.49%)	222 (86.38%)	
Yes	132 (17.51%)	63 (13.62%)	
N/A	14	5	
PGS			0.448
Mean (SD)	− 0.73 (0.48)	− 0.75 (0.43)	

*N* number of participants, *N/A* not applicable, *PGS* polygenic score, *RTP* return to play, *RTPi* return to play initiation, *SCAT* Sport Concussion Assessment Tool, *SD* standard deviation

**Fig. 2:**
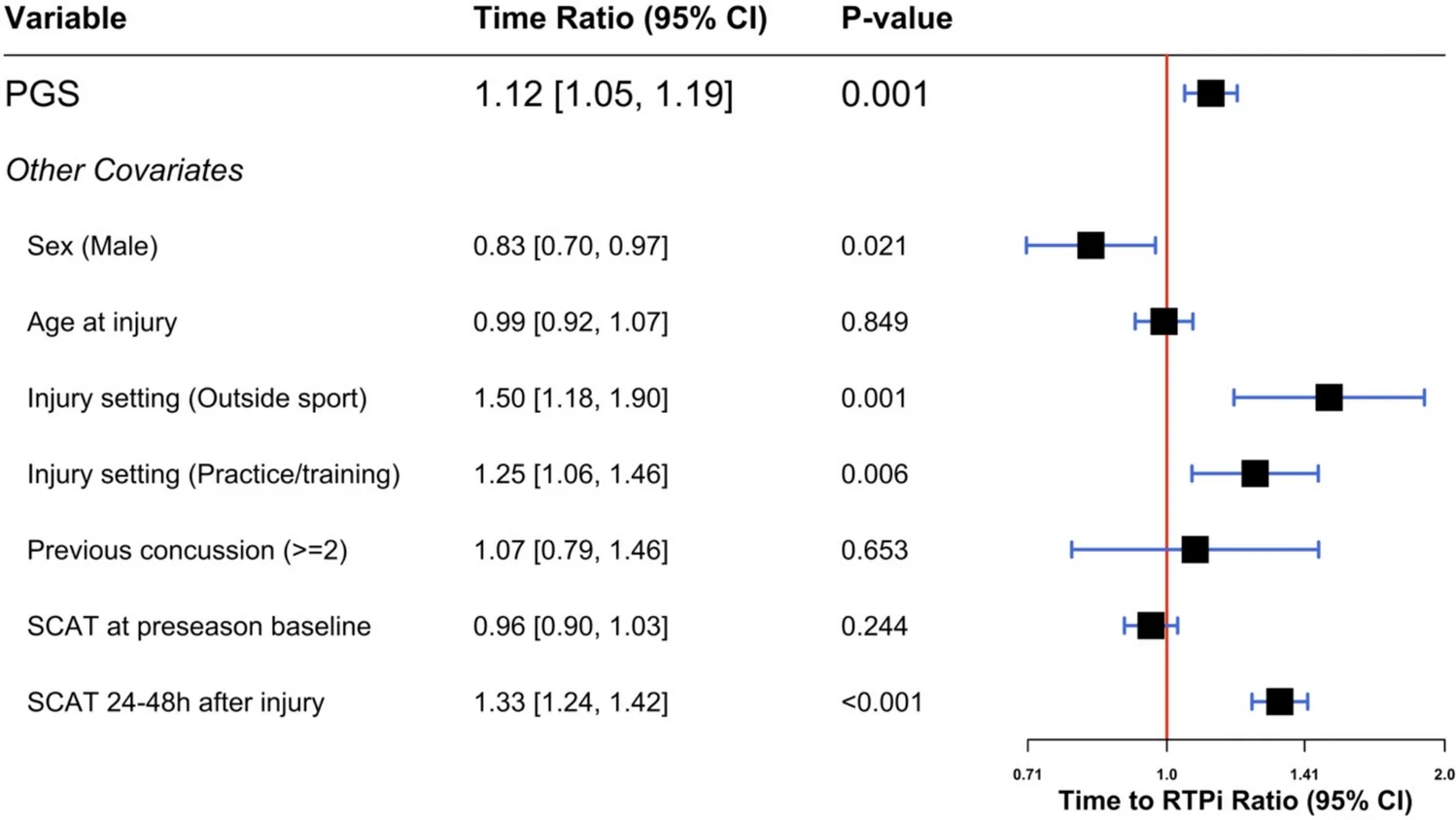
Forest plot for time-to-RTPi outcome in the validation cohort. The model was adjusted for the first 5 principal components (PCs) of genetic ancestries. Continuous variables (PGS, age at injury, preseason baseline SCAT symptom severity score, and post-injury 24–48-h SCAT symptom severity score) were standardized. Each variable is represented with its corresponding time ratio defined as the multiplicative change in median time to RTPi per one-unit increase in the factor and 95% confidence interval (CI). *p*-Values were calculated using Wald tests on the log-logistic AFT regression coefficients. *AFT* accelerated failure time, *PGS* polygenic score, *RTPi* return to play initiation, *SCAT* Sport Concussion Assessment Tool

The effect of PGS, alongside other covariates, on the time-to-RTPi outcome in the validation cohort is shown in Fig. 2. Higher PGS was significantly associated with a longer time to reach RTPi (*p* = 0.001), and each standard deviation increase in PGS was associated with a 12% increase in time to achieve RTPi. Additional variables significantly associated with longer time to RTPi included being female, sustaining an injury during practice/training or outside sport versus during competition, and having a worse post-injury 24–48-h SCAT symptom severity score. Model performance comparisons based on the concordance index (*C*-index) showed that adding genetic ancestry information (first five PCs) improved prediction beyond demographic and injury-related factors (from 0.69 to 0.70) and incorporating PGS further increased the *C*-index to 0.71, resulting in a total improvement of 0.02.

**Fig. 3:**
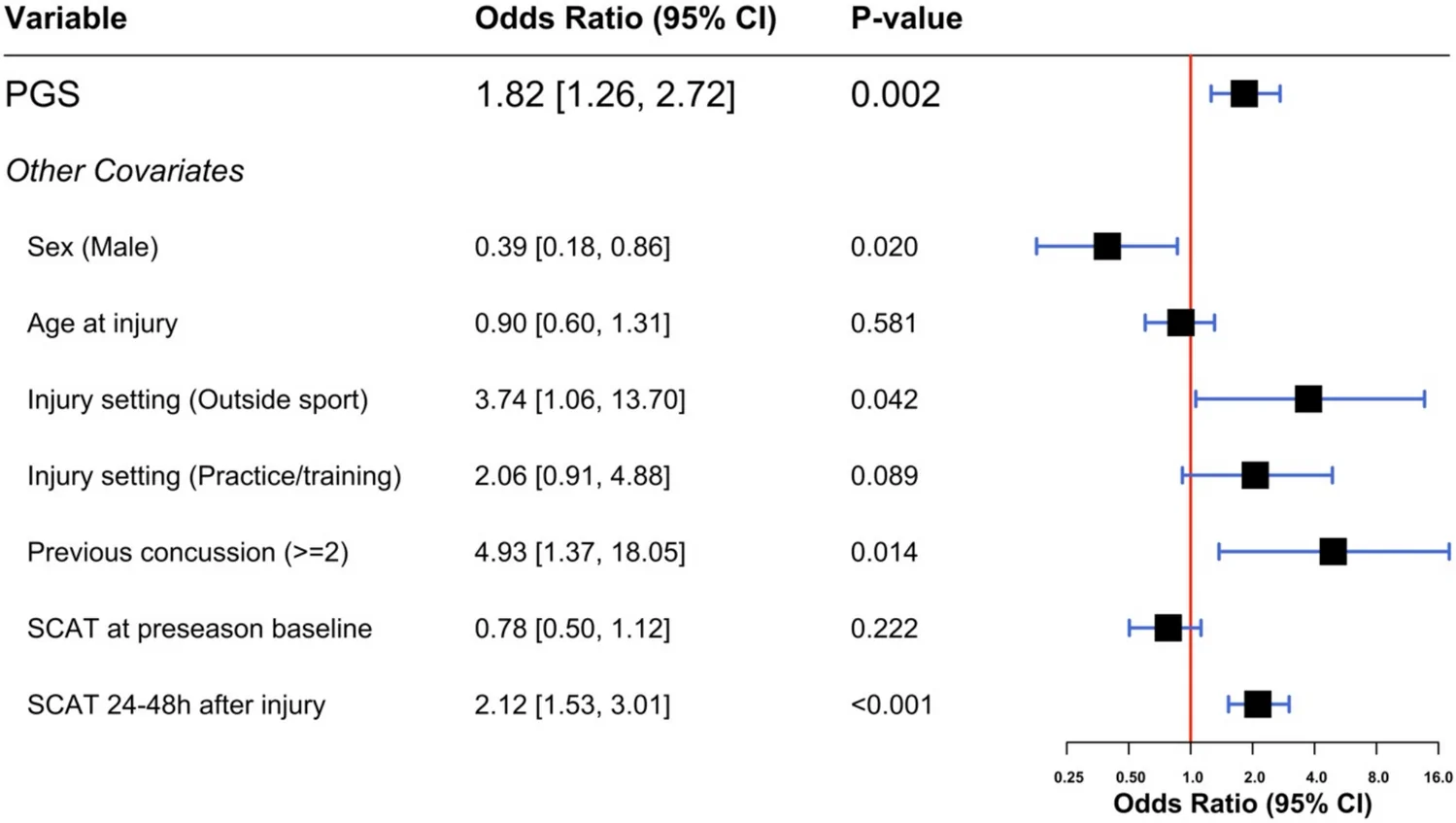
Forest plot for slow RTPi (≥ 14 days) outcome in the validation cohort. The model was adjusted for the first 5 principal components (PCs) of genetic ancestries. Continuous variables (PGS, age at injury, preseason baseline SCAT symptom severity score, and post-injury 24–48-h SCAT symptom severity score) were standardized. Each variable is represented with its corresponding odds ratio and 95% confidence interval (CI). *p* Values were calculated using Wald tests on the logistic regression coefficients. *PGS* polygenic score, *RTPi* return to play initiation, *SCAT* Sport Concussion Assessment Tool

**Fig. 4:**
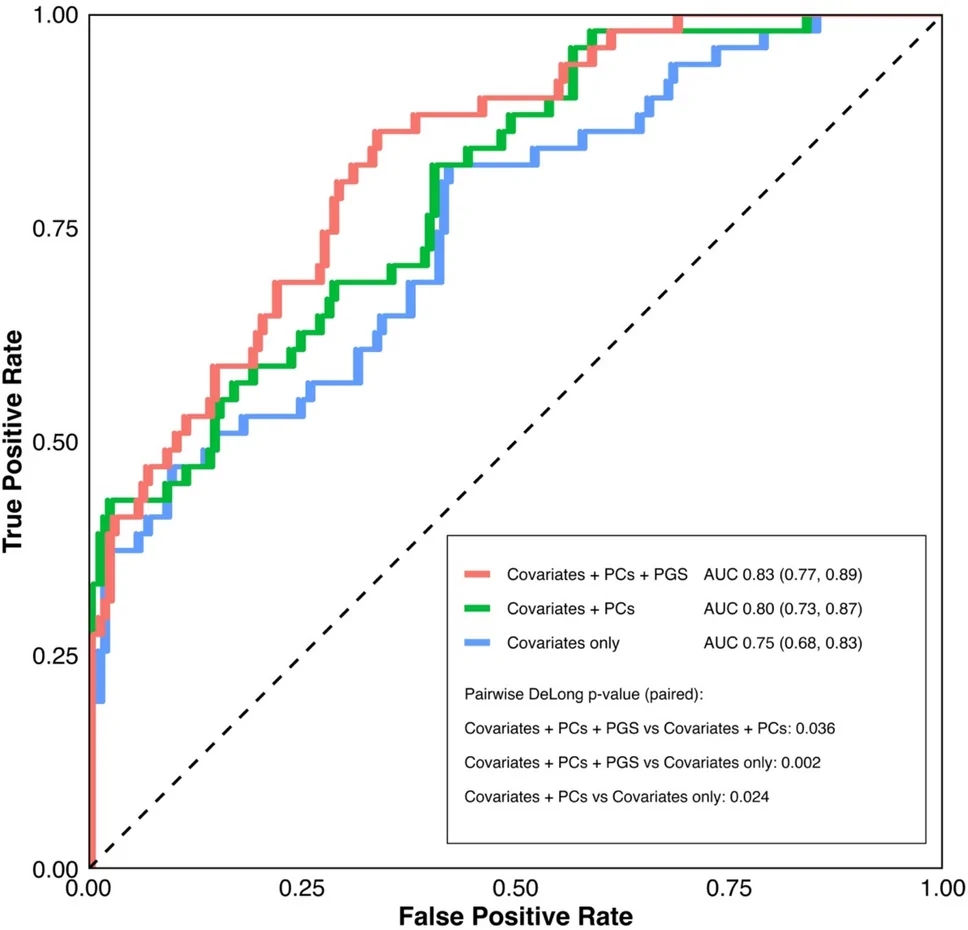
ROC curves for predicting slow RTPi (≥ 14 days) outcome in the validation cohort. The red curve shows the model with PGS, the first 5 PCs of genetic ancestries, and covariates; green: the first 5 PCs and covariates; blue: covariates only. Covariates include age at injury, sex, injury setting, diagnosed concussion history (≥ 2 vs ≤ 1), and SCAT scores at baseline and 24–48 h post-injury. AUC values with 95% confidence intervals (DeLong method) for each model and pairwise DeLong *p* values for correlated ROC curve comparisons are shown. *AUC* area under the ROC, *PC* principal component, *PGS* polygenic score, *ROC* receiver operating characteristic curve, *RTPi* return to play initiation, *SCAT* Sport Concussion Assessment Tool

To evaluate whether genetic predisposition contributes to prolonged recovery, we examined the association between PGS and the odds of slow RTPi in the validation cohort (Fig. 3). We found that each standard deviation increase in the PGS was significantly associated with an 82% higher odds of slow RTPi recovery (*p* = 0.002). Additionally, being female, sustaining an injury during practice/training (marginally significant) or outside sport, having two or more previous concussions, and experiencing a worse post-injury 24–48-h SCAT symptom severity score were also significantly associated with higher odds of slow RTPi recovery. Model performance, assessed using the area under the ROC curve (AUC), improved from 0.75 (95% CI 0.68–0.83) for covariates only to 0.80 (95% CI 0.73–0.87) with the addition of genetic ancestry information and further to 0.83 (95% CI 0.77–0.89) with the inclusion of PGS (Fig. 4). All pairwise DeLong comparisons were statistically significant (*p* values shown in Fig. 4).

We also assessed the association between PGS and RTPi recovery outcomes across racial subgroups (Supplementary Table 2, ESM). For time to RTPi, PGS was significantly associated with longer recovery in European American (time ratio 1.15, *p* = 0.001) and African American participants (time ratio 1.17, *p* = 0.038), but not in the Other subgroup (time ratio 1.00, *p* = 0.986). Similarly, for slow RTPi, PGS was significantly associated with higher odds of slow RTPi recovery in European American participants (OR 2.38, *p* = 0.001), whereas no significant associations were observed in African American (OR 0.98, *p* = 0.972) or Other participants (OR 1.31, *p* = 0.778). The lack of significance for some subgroups was likely due to the small sample sizes, particularly the limited number of individuals in the slow RTPi recovery group (*n*) and the overall sample size (*N*) within that racial category; these subgroup analyses are therefore considered exploratory. Alternative allele frequencies (AAFs) of SNPs used in PGS construction for European and African ancestries, as well as in CARE samples, are presented in Supplementary Table 3 (see ESM).

As a secondary analysis, we evaluated whether genetic predisposition contributes to unrestricted RTP variability by testing the effects of the developed PGS on time to RTP and slow RTP (≥ 28 days) outcomes in the validation cohort. No significant association was observed between PGS and either time to RTP (*p* = 0.304; Supplementary Fig. 1, ESM) or slow RTP (≥ 28 days; *p* = 0.962; Supplementary Fig. 2, ESM).

**Fig. 5:**
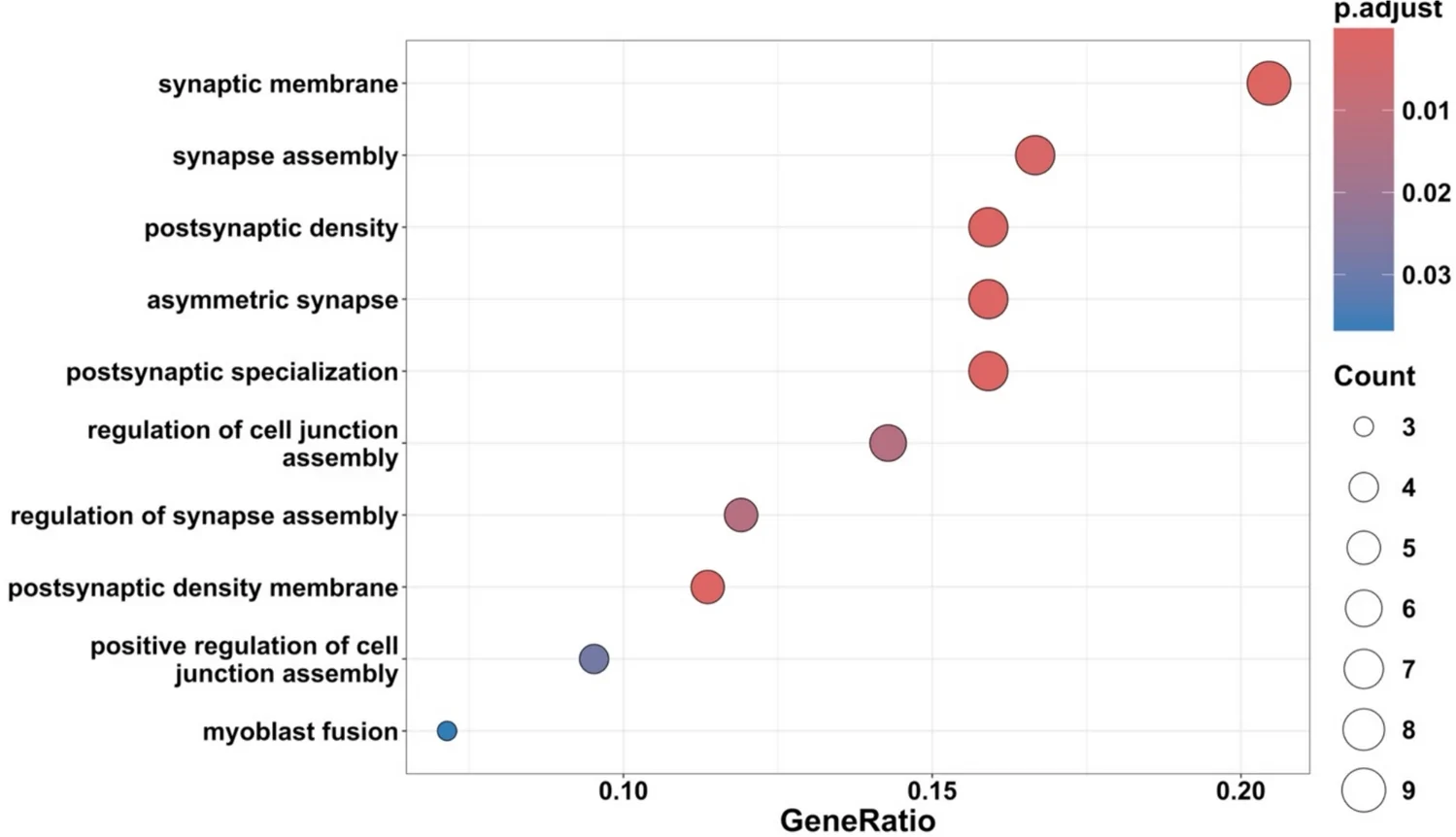
Gene Ontology (GO) enrichment analysis for the SNPs used in PGS construction. The *X*-axis represents the GeneRatio (the proportion of associated genes mapping to each GO term), and the *Y*-axis lists the top 10 significantly enriched biological terms. Each circle represents an enriched GO term, with its size corresponding to the number of associated genes contributing to the enrichment. The circle color indicates the adjusted *p* value (*p*.adjust), with warmer colors (red) denoting lower *p* values and cooler colors (blue) denoting higher *p*-values. *PGS* polygenic score, *SNPs* single-nucleotide polymorphisms

To further investigate the biological mechanisms underlying concussion recovery, we performed GO enrichment analysis on the SNPs utilized in PGS construction. This analysis revealed several biological processes and cellular components relevant to concussion recovery (Fig. 5). Key enriched terms, such as synaptic membrane, synapse assembly, and postsynaptic density, emphasized the role of synaptic organization and function in restoring neural communication after injury. Processes like regulation of synapse assembly and regulation of cell junction assembly highlighted the mechanisms involved in synaptic repair and stabilization, essential for neuronal network recovery. Additionally, terms such as asymmetric synapse and postsynaptic specialization underscored the importance of precise synaptic architecture in reestablishing effective neurotransmission. These findings suggested that the SNPs incorporated in the PGS capture pathways critical for synaptic signaling, structural integrity, and neuronal repair, all of which were integral to the recovery process following concussion. Notably, among the selected SNPs, we found an A > G variant (rs762636) in the promoter region of the *F7* gene, which encodes coagulation factor VII, to be associated with longer recovery times (*p* = 5.119 × 10^–5^), suggesting a potential role for vascular repair mechanisms in concussion recovery.

## Discussion

This study investigated the genetic contributions to concussion recovery outcomes, including time to RTPi and slow RTPi. The major conclusion of the current study is that genetic predisposition affects concussion recovery. This conclusion is based on the significant association of a PGS with RTPi, where each standard deviation increase in PGS corresponds to a 12% longer time to RTPi (*p* = 0.001). Moreover, the genetic variants incorporated in the PGS implicate relevant biological processes, such as neuronal repair and re-establishment of proper neuronal communication, which are likely critical in the recovery process. The significant association between PGS and slow RTPi (OR 1.82, *p* = 0.002) further highlights its value in identifying individuals at risk for delayed recovery. Prolonged RTPi recovery is a critical clinical metric, as it identifies individuals who may face extended disruptions in cognitive, physical, and emotional function. Identifying these individuals early through tools like the PGS could allow for more targeted interventions, potentially reducing the burden of prolonged recovery.

Our finding that the PGS and genetic ancestry improved risk prediction performance for time to RTPi suggests the PGS plus covariates model effectively distinguishes individuals with earlier versus later recovery timing. The enhancement was more pronounced for the binary slow RTPi outcome, where the AUC increased from 0.75 (covariates only) to 0.83 with the addition of PGS and genetic ancestry data, indicating a substantial boost in discriminative power. An AUC exceeding 0.8 is generally interpreted as indicative of strong discrimination [14, 34], supporting the potential utility of integrating genetic information into predictive models for identifying individuals at risk of prolonged recovery.

The biological insights gained from our analysis highlight the complex interplay of neural and vascular processes in concussion recovery. The enrichment of synaptic organization, signaling, and neuronal repair processes supports the involvement of neuronal network reestablishment, a phenomenon paralleled in preclinical models [35, 36]. For example, Giza and Prins demonstrated that young rats with mTBI exhibited acute alterations in gene expression governing metabolic function, neurotransmission, neuroplasticity, and growth factor signaling, providing a potential mechanistic basis for our observed synaptic pathway involvement [35]. Similarly, Chapman et al. showed that repetitive head impacts can induce functional changes in neuroplasticity without overt cell death, underscoring the importance of synaptic resilience and remodeling in recovery [36]. Concurrently, we found a promoter variant in the *F7* gene associated with prolonged recovery, which suggests that vascular repair may also play a meaningful role. The *F7* gene encodes coagulation factor VII, which is a key initiator of the extrinsic coagulation pathway. This is a potentially relevant finding because hemostatic/platelet dysfunction has been reported after mTBI/concussion, supporting a link between vascular–coagulation responses and post-injury recovery [37]. Moreover, prior work in isolated TBI has implicated reduced factor VII activity and *F7* genetic variation in coagulopathy and progressive hemorrhagic injury, providing a plausible pathway through which *F7* regulatory variation could influence recovery trajectories [38]. This dual involvement underscores the interconnected nature of neuronal and vascular healing mechanisms. Together, these results offer valuable insights into the biological mechanisms underlying concussion recovery and underscore the relevance of the selected SNPs for PGS development.

Our study has several key strengths. First, we utilized a well-characterized discovery cohort and validated our findings in an independent cohort, enhancing the generalizability and robustness of our results. Second, we employed a robust variable selection method to refine polygenic score construction. Due to its robustness, this method reduces false identification and improves the reliability of SNP selection in the context of high-dimensional genomic data [39, 40]. Third, the use of PGS allowed us to aggregate the effects of multiple SNPs, making it possible to detect meaningful genetic associations with a smaller sample size. Fourth, the integration of Gene Ontology enrichment analysis linked the PGS-associated SNPs to biologically relevant pathways, thereby enhancing the translational potential of our findings.

This study also has some limitations. The CARE Consortium participants fall in a narrow age range by design, including collegiate athletes and military cadets. While this ensures homogeneity and precise effect estimates within this high-risk group, the findings may not generalize to other populations, such as younger athletes, older individuals experiencing concussion, or non-athletes. Any extrapolation to other populations should therefore be considered hypothesis-generating, as biological mechanisms of recovery may overlap across groups. However, differences in age distribution, comorbidities, injury mechanisms, and clinical care pathways require external replication and potential recalibration before clinical application. In addition, our discovery GWAS sample size was modest (*N* = 768), which limits statistical power to detect small single-SNP effects. While this reduces power for single-locus discovery, PGS are designed to aggregate many SNPs of small effect into a composite measure and therefore have the potential to capture polygenic signal even when individual SNP associations do not reach genome-wide significance [41, 42]. Additionally, as noted, there were 14 out of 768 participants in the discovery cohort and 5 out of 262 in the validation cohort who lacked both recorded RTPi and RTP data by the end of follow-up. Therefore, their data may not fully represent final recovery trajectories. Nevertheless, AFT models effectively handled these cases as censored data by leveraging the available partial information, ensuring valid inference and reliable conclusions. Additionally, we tested the developed PGS on a related concussion recovery outcome, time to RTP and slow RTP (≥ 28 days), in the validation cohort, but observed no significant associations. Unlike RTPi, unrestricted RTP decisions are often influenced by non-clinical considerations, such as institutional protocols, team expectations, and physical readiness, and emerging evidence suggests that certain supplements and nutrients may also influence recovery [43]; together, these factors may obscure genetic contributions. Notably, while our primary outcomes focus on time to RTPi and unrestricted RTP, we acknowledge that investigating multidomain functional measures, such as motor/sensorimotor function, cognitive performance, and neurobehavioral outcomes, could also be valuable in future studies [44]. Nonetheless, our use of independent validation, robust PGS construction, and Gene Ontology enrichment analysis strengthens the reliability and translational potential of our findings.

In the future, validated polygenic scores could be used as an adjunct to standard clinical assessment to support individualized concussion management. For example, a PGS associated with slower recovery could help identify individuals who may benefit from closer follow-up, earlier referral for targeted rehabilitation, and a more conservative progression of activity. Any clinical use would require replication in larger, diverse cohorts, calibration across ancestries, and demonstration that PGS adds predictive value beyond established clinical risk factors.

## Conclusions

The PGS we developed showed significant associations with time to RTPi, highlighting the clinical relevance of genetic contributions to concussion recovery. To our knowledge, this is the first study to construct and validate a PGS specifically for concussion recovery outcomes. The clinical relevance of these findings, while limited in current healthcare practice, warrants further investigation toward a more personalized approach to concussion management.

## Supplementary Information

Below is the link to the electronic supplementary material.

Supplementary file1 (DOCX 1478 KB)

## Declarations

### Data availability

The genotyping and GWAS data generated in this study were deposited in the Federal Interagency Traumatic Brain Injury Research (FITBIR) Informatics System under the study name: Concussion Assessment, Research and Education (CARE) Consortium. The dataset names and IDs are: CARE1_GWAS_final_n546_Biomarker, ID 17733; and CARE2_GWAS_Biomarker, ID 17972.
