# Supplementary material for: Polygenic Score Identifies Athletes at Increased Risk for Slower Recovery After Sport-Related Concussion: A Concussion Assessment, Research, and Education (CARE) Consortium Study
Source: Sports Med. 2026 Jun 30;56(10):2583–95. doi: 10.1007/s40279-026-02477-6 (PMC13615007; doi:10.1007/s40279-026-02477-6)
Supplement: Supplementary file 1 [file 40279_2026_2477_MOESM1_ESM.docx]

**Supplementary Data**

| Cohort | Race | Female, n (%) | Male, n (%) |
| --- | --- | --- | --- |
| Discovery | European American | 242 (31.51%) | 304 (39.58%) |
| Discovery | African American | 26 (3.39%) | 99 (12.89%) |
| Discovery | Other | 48 (6.25%) | 49 (6.38%) |
| Totals |  | **316 (41.15%)** | **452 (58.85%)** |
| Validation | European American | 50 (19.08%) | 117 (44.66%) |
| Validation | African American | 10 (3.82%) | 46 (17.56%) |
| Validation | Other | 8 (3.05%) | 31 (11.83%) |
| Totals |  | **68 (25.95%)** | **194 (74.05%)** |

**Supplementary Table 1.** Sex-by-racial group composition of the discovery and validation cohorts. Counts and percentages of female and male participants are shown within each racial subgroup for the discovery and validation cohorts. Percentages are calculated within each cohort.

|  | Time-to-RTPi | | |  | Slow RTPi | | |
| --- | --- | --- | --- | --- | --- | --- | --- |
| Race | N | PGS Effect  (Time Ratio, 95% CI) | P-value |  | n/N (%) | PGS Effect (Odds Ratio, 95% CI) | P-value |
| European American | 167 | 1.15 (1.06, 1.25) | **0.001** |  | 40/164 (24.39%) | 2.38 (1.41, 4.02) | **0.001** |
| African American | 56 | 1.17 (1.01, 1.36) | **0.038** |  | 10/56 (17.86%) | 0.98 (0.30, 3.22) | 0.972 |
| Other | 39 | 1.00 (0.85, 1.18) | 0.986 |  | 13/37 (35.14%) | 1.31 (0.20, 8.63) | 0.778 |

**Supplementary Table 2.** Effects of PGS on time-to-RTPi (left panel) and slow RTPi (right panel) outcomes in the validation cohort, stratified by racial subgroup. This table summarizes the PGS effects on RTPi outcomes, stratified by self-identified racial subgroups (European American, African American, and Other). The PGS effect on the time-to-RTPi outcome was modeled using a log-logistic AFT model, while the slow RTPi outcome (≥ 14 days) was modeled using logistic regression. Both models were adjusted for the same covariates used in PGS construction. Bold text indicates statistical significance at p < 0.05. For time-to-RTPi, N represents the total number of individuals in each racial subgroup included in the analysis. For slow RTPi, n/N (%) denotes the number (n) and percentage of individuals who experienced slow recovery out of the total number (N) in each subgroup.

| SNP | Chromosome | Position | Nearest Gene | Reference AAF (European) | Reference AAF  (African) | CARE Samples AAF |
| --- | --- | --- | --- | --- | --- | --- |
| rs4661679 | 1 | 16321010 | SRARP | 0.385 | 0.539 | 0.422 |
| rs77318967 | 1 | 237346821 | RYR2 | 0.081 | 0.065 | 0.087 |
| rs58546652 | 1 | 247601778 | NLRP3 | 0.646 | 0.497 | 0.597 |
| rs73931632 | 2 | 49501531 | FSHR | 0.122 | 0.140 | 0.109 |
| rs6545115 | 2 | 49666647 | FSHR | 0.882 | 0.842 | 0.864 |
| rs56214913 | 2 | 55320565 | RTN4 | 0.052 | 0.035 | 0.055 |
| rs12477144 | 2 | 71981934 | DYSF | 0.293 | 0.035 | 0.254 |
| rs77468690 | 2 | 225495128 | CUL3 | 0.065 | 0.004 | 0.055 |
| rs17061598 | 3 | 59958728 | FHIT | 0.080 | 0.122 | 0.101 |
| rs77563983 | 3 | 60123450 | FHIT | 0.066 | 0.013 | 0.057 |
| rs11707687 | 3 | 106072768 | CBLB | 0.445 | 0.238 | 0.424 |
| rs34413884 | 3 | 139841076 | CLSTN2 | 0.495 | 0.132 | 0.434 |
| rs11927704 | 3 | 188517106 | LPP | 0.320 | 0.207 | 0.295 |
| rs3927450 | 4 | 20265910 | SLIT2 | 0.352 | 0.352 | 0.309 |
| rs4859674 | 4 | 77376813 | SHROOM3 | 0.733 | 0.604 | 0.744 |
| rs67445745 | 4 | 100323878 | ADH7 | 0.259 | 0.134 | 0.209 |
| rs11214021 | 4 | 107462552 | LINC02173 | 0.038 | 0.147 | 0.052 |
| rs7668281 | 4 | 162386388 | FSTL5 | 0.638 | 0.822 | 0.664 |
| rs61751836 | 5 | 11167774 | CTNND2 | 0.039 | 0.089 | 0.056 |
| rs11242558 | 5 | 104889755 | RAB9BP1 | 0.366 | 0.569 | 0.403 |
| rs1283903 | 6 | 69738032 | ADGRB3 | 0.148 | 0.053 | 0.152 |
| rs73753646 | 6 | 87735594 | HTR1E | 0.227 | 0.303 | 0.249 |
| rs72981049 | 6 | 104007856 | HACE1 | 0.280 | 0.050 | 0.245 |
| rs35934101 | 6 | 137635461 | IFNGR1 | 0.035 | 0.157 | 0.055 |
| rs74472841 | 7 | 1737867 | ELFN1 | 0.112 | 0.076 | 0.089 |
| rs76373479 | 7 | 30230870 | MTURN | 0.120 | 0.055 | 0.102 |
| rs55828133 | 7 | 35583137 | HERPUD2 | 0.128 | 0.313 | 0.172 |
| rs10085609 | 7 | 146856717 | CNTNAP2 | 0.065 | 0.258 | 0.085 |
| rs2061685 | 8 | 107633768 | OXR1 | 0.046 | 0.081 | 0.054 |
| rs7460111 | 8 | 135486254 | ZFAT | 0.081 | 0.057 | 0.064 |
| rs10812688 | 9 | 27912943 | LINGO2 | 0.325 | 0.157 | 0.318 |
| rs10116555 | 9 | 34407423 | FAM219A | 0.209 | 0.353 | 0.202 |
| rs12341244 | 9 | 104034524 | PLPPR1 | 0.389 | 0.418 | 0.405 |
| rs482468 | 10 | 27671644 | PTCHD3 | 0.692 | 0.576 | 0.661 |
| rs34196631 | 10 | 129276845 | DOCK1 | 0.137 | 0.025 | 0.133 |
| rs13343175 | 11 | 6477455 | TRIM3 | 0.185 | 0.332 | 0.204 |
| rs7342213 | 11 | 97758518 | CNTN5 | 0.118 | 0.117 | 0.107 |
| rs61913072 | 12 | 1409069 | ERC1 | 0.120 | 0.004 | 0.080 |
| rs12231866 | 12 | 24245753 | SOX5 | 0.405 | 0.274 | 0.398 |
| rs1554839 | 12 | 81020376 | PTPRQ | 0.311 | 0.479 | 0.341 |
| rs11108839 | 12 | 97534882 | NEDD1 | 0.072 | 0.280 | 0.087 |
| rs762636 | 13 | 113759526 | F7 | 0.220 | 0.063 | 0.195 |
| rs1951679 | 14 | 32723703 | RNU6-7 | 0.103 | 0.268 | 0.124 |
| rs2623990 | 15 | 73552174 | NEO1 | 0.082 | 0.193 | 0.083 |
| rs59447674 | 15 | 95295647 | MCTP2 | 0.471 | 0.738 | 0.503 |
| rs11649040 | 16 | 373951 | AXIN1 | 0.268 | 0.420 | 0.288 |
| rs11320223 | 16 | 13206859 | SHISA9 | 0.041 | 0.126 | 0.059 |
| rs35336534 | 22 | 17622618 | HDHD5 | 0.258 | 0.129 | 0.183 |
| rs5753146 | 22 | 30842095 | SEC14L3 | 0.605 | 0.915 | 0.672 |

**Supplementary Table 3** SNPs used in PGS construction (chromosome, base‑pair position, nearest gene), with alternative allele frequencies (AAFs) in reference European and African Ancestries, and in CARE samples. Reference European and African ancestry AAF are from the 1000 Genomes Project. The CARE Samples include all individuals from the discovery and validation cohorts used in this study.

**Supplementary Figure 1** Forest plot for time-to-RTP outcome in the validation cohort**.** The model was adjusted for the first 5 principal components (PCs) of genetic ancestries. Continuous variables (PGS, age at injury, preseason baseline SCAT symptom severity score, and post-injury 24–48-hour SCAT symptom severity score) were standardized. Each variable is represented with its corresponding time ratio and 95% confidence interval (CI). P-values were calculated using Wald tests on the log-logistic AFT regression coefficients. PGS, polygenic score; SCAT, Sport Concussion Assessment Tool; RTP, return to play.

**Supplementary Figure 2** Forest plot for slow RTP (≥ 28 days) outcome in the validation cohort**.** The model was adjusted for the first 5 principal components (PCs) of genetic ancestries. Continuous variables (PGS, age at injury, preseason baseline SCAT symptom severity score, and post-injury 24–48-hour SCAT symptom severity score) were standardized. Each variable is represented with its corresponding odds ratio and 95% confidence interval (CI). P-values were calculated using Wald tests on the logistic regression coefficients. PGS, polygenic score; SCAT, Sport Concussion Assessment Tool.
